# Cellular Contractility Profiles of Human Diabetic Corneal Stromal Cells

**DOI:** 10.1155/2021/9913210

**Published:** 2021-06-04

**Authors:** Thi N. Lam, Sarah E. Nicholas, Alexander Choi, Jian-Xing Ma, Dimitrios Karamichos

**Affiliations:** ^1^Dean McGee Eye Institute, Oklahoma University Health Sciences Center, 608 Stanton L Young Blvd, Oklahoma City, OK 73104, USA; ^2^North Texas Eye Research Institute, University of North Texas Health Science Center, 3500 Camp Bowie Blvd, Fort Worth, TX 76107, USA; ^3^Department of Pharmaceutical Sciences, University of North Texas Health Science Center, 3500 Camp Bowie Blvd, Fort Worth, TX 76107, USA; ^4^Department of Physiology, University of Oklahoma Health Sciences Center, 940 Stanton L. Young, Oklahoma City, OK, USA; ^5^Harold Hamm Oklahoma Diabetes Center, 1000 N Lincoln Blvd, Oklahoma City, OK, USA; ^6^Department of Pharmacology and Neuroscience, University of North Texas Health Science Center, 3500 Camp Bowie Blvd, Fort Worth, TX 76107, USA

## Abstract

Diabetic keratopathy is a corneal complication of diabetes mellitus (DM). Patients with diabetic keratopathy are prone to developing corneal haze, scarring, recurrent erosions, and significant wound healing defects/delays. The purpose of this study was to determine the contractility profiles in the diabetic human corneal stromal cells and characterize their molecular signatures. Primary human corneal fibroblasts from healthy, Type 1 DM (T1DM), and Type 2 DM (T2DM) donors were cultured using an established 3D collagen gel model. We tracked, measured, and quantified the contractile footprint over 9 days and quantified the modulation of specific corneal/diabetes markers in the conditional media and cell lysates using western blot analysis. Human corneal fibroblasts (HCFs) exhibited delayed and decreased contractility compared to that from T1DMs and T2DMs. Compared to HCFs, T2DMs demonstrated an initial downregulation of collagen I (day 3), followed by a significant upregulation by day 9. Collagen V was significantly upregulated in both T1DMs and T2DMs based on basal secretion, when compared to HCFs. Cell lysates were upregulated in the myofibroblast-associated marker, *α*-smooth muscle actin, in T2DMs on day 9, corresponding to the significant increase in contractility rate observed at the same time point. Furthermore, our data demonstrated a significant upregulation in IGF-1 expression in T2DMs, when compared to HCFs and T1DMs, at day 9. T1DMs demonstrated significant downregulation of IGF-1 expression, when compared to HCFs. Overall, both T1DMs and T2DMs exhibited increased contractility associated with fibrotic phenotypes. These findings, and future studies, may contribute to better understanding of the pathobiology of diabetic keratopathy and ultimately the development of new therapeutic approaches.

## 1. Introduction

Diabetes mellitus (DM) is a major public health problem and one of the most prevalent chronic diseases worldwide [1]. DM continues to rise in numbers and significance, affecting epidemic proportions globally [2]. In 2014, the World Health Organization (WHO) stated that approximately 422 million adults worldwide were suffering from DM, almost doubling from 4.7% in 1980 to 8.5%. The number of people with DM is projected to further increase since the disease is not only predominate in adults but children as well [3–5].

The etiology of the two most common DM types, Type 1 and Type 2, is a complex interplay of genetics, lifestyle preferences, and environmental factors [6]. Type 1 DM (T1DM) is a consequence of autoimmune beta cell destruction, which leads to insulin deficiency, and accounts for approximately 10% of cases, primarily children and young adults. On the other hand, Type 2 DM (T2DM) accounts for 90% of cases and generally forms part of a metabolic interaction, which is defined by insulin resistance, cardiovascular risk factors, and obesity [7, 8]. DM-related ocular complications are one of the leading causes of adult blindness [9, 10]. Both diabetic retinopathy and diabetic keratopathy are eminent risk factors for visual deterioration in DM patients, resulting in more than 20,000 new cases of blindness every year [11, 12].

Diabetic keratopathy-related abnormalities may include, but are not limited to, stromal edema, deposition of advanced glycation end products, decreased corneal sensitivity, recurrent corneal erosions, delayed corneal wound healing, and neurotrophic corneal ulcers [13–15]. When the corneal epithelium and stromal layers are affected, it is vital that the stroma and epithelium reattach or further tissue defects can develop which often reoccur. Furthermore, under hyperglycemic conditions, increased reactive oxygen species (ROS), advanced glycation end product (AGE) immune reactivity, and mitochondrial injury in the cornea have all been reported [16]. The diabetic corneal epithelium and stroma are resistant to traditional treatment regimens, due to the fact that hyperglycemia significantly changes the structure and function of both layers, resulting in altered levels of cell proliferation, weak barrier function, abnormal collagen deposition, and collagen crosslinking [17–21]. Such alterations expose diabetic patients to higher susceptibility of corneal infections, stromal ulcerations, erosion, scarring, and ultimately vision loss [22].

The human corneal stroma is rich in type I collagen, but also contains type V and type VI collagens [23, 24]. The cornea harbors dormant keratocytes that, when activated by pathological processes, can differentiate into active fibroblasts and subsequently myofibroblasts which deposit type III collagen. Type III collagen is critical upon injury/trauma and the subsequent wound healing cascade. This transformation process is critical to corneal wound closure and contraction. Unfortunately, myofibroblasts are responsible for the pathologic processes of corneal haze and scarring [25–27].

In the present study, we utilized a detached, free-floating 3D collagen gel model, in order to determine the contractile “signatures” of the diabetic corneal stroma cells [28]. We further investigated the modulation of established corneal and diabetic markers as a function of contraction. Overall, our study highlights the importance of utilizing 3D collagen gels to examine cellular-extracellular matrix (ECM) interactions in the context of diabetic keratopathy. To our knowledge, this is the first study utilizing 3D free-floating collagen gels to investigate the diabetic corneal stroma.

## 2. Materials and Methods

### 2.1. Ethics and Inclusion Criteria

All parts of this study adhere to the Declaration of Helsinki ethical principles. Institutional review board approval was received prior to initiation of all experiments defined in this study. Human corneal samples were obtained from the National Development and Research Institute (NDRI) and the Oklahoma Lions Eye Bank. The North Texas Regional Institutional Review Board (IRB) was notified, and appropriate permission was obtained prior to initiations of experimental procedures (#2020-30). All diabetic samples adhered to strict inclusion/exclusion criteria where DM donors with a clinical diagnosis of T1DM or T2DM were included, only if no other systemic and unrelated diseases or ocular pathology existed. The healthy control group was comprised of corneas isolated from cadavers with no history of ocular trauma or systemic diseases. The cause of death for healthy controls ranged from accidental to non-DM-related diseases (blunt force trauma, head trauma, end-stage renal disease, acute segment elevation myocardial infarction, subarachnoid hemorrhage, and cardiac arrest). In this study, a total of 8 diabetic donor corneal samples (4 donors for each T1DM and T2DM) and 4 healthy age-matched control samples were analyzed. The average age range for donors included in this study was 55-59 years of age, and the duration of DM was from 3 to 30 years. We observed no data bias, based on the age or duration of DM.

### 2.2. Primary Cell Isolation and Cultures

Healthy and DM corneas were obtained and processed. Stromal cells were isolated as previously described [29, 30]. Briefly, both the endothelium and epithelium were removed from the stroma by scraping briefly with a razor blade; furthermore, the stroma was cut into ~2 mm × 2 mm pieces. The corneal pieces were then allowed to adhere to the bottom of a T75 flask for 30 minutes at 37°C before adding 10% Fetal Bovine Serum (FBS) Eagle's Minimum Essential Media (EMEM) and 1% antibiotic/antimycotic (Gibco® Antibiotic-Antimycotic, Life Technologies). At approximately 80% confluency, the explants were passaged in 10% FBS in EMEM and 1% antibiotic for further expansion. All experiments were executed using cells between passages 3 and 7.

### 2.3. Collagen Contraction Assay

Rat-tail collagen type I (Advanced Biomatrix, San Diego, California) was mixed with EMEM on ice with 125 *μ*L EMEM per 1 mL collagen. The pH was then adjusted to pH 7–8 with 1 M NaOH. Healthy human corneal fibroblasts (HCFs), T1DMs, or T2DMs were added at a concentration of 5 × 10^5^ and mixed slowly to avoid air bubbles. This mixture was plated in a 12-well plate at 1 mL per well and incubated in 37°C for 30 min to promote solidification. After congealing, 1 mL of 10% FBS EMEM was added on top of the gels (Karamichos et al., 2009; Lyon et al., 2015). The collagen gels were released after 48 h of incubation by running a sterile blade around the edges of the well. The contraction of the collagen gels was monitored by measuring the gel diameter daily for 9 days. The area of the gel was quantified using ImageJ software. Calculating the contractility rate, the average area of the gels was subtracted from the average area on day 0 and divided by the number of days that had passed.

### 2.4. Western Blot Analysis

Western blots were performed on cell lysates and conditional media collected from all experiments, as per our previously optimized protocol [31]. Preparation of cell lysates was initiated by using RIPA buffer (50 mM Tris, pH 8, 150 mM NaCl, 1% Triton X-100, 0.1% SDS, and 1% sodium deoxycholate) containing protease and phosphatase inhibitors (Sigma Aldrich; St. Louis, MO), followed by brief incubation and centrifugation and stored at -20°C until further processing. Total protein content within conditioned media and cell lysates was measured using a BCA assay (Thermo Scientific, Rockford, IL, USA). Samples were then normalized to the sample containing the lowest protein content, thereby enabling equal loading onto the gel. Media samples were run on a 4–20% precast polyacrylamide gradient gel at 130 V for 1.5 h then transferred to a nitrocellulose membrane on ice at 100 V for 1 h. The membrane was blocked in a 5% milk solution in Tris-buffered solution with Tween 20 (TBST) for 1 h, followed by overnight incubation in a cold room with 1 : 1000 primary antibodies. Antibodies used include collagen I (ab34710; Abcam, Cambridge, MA, USA), collagen III (ab7778; Abcam, Cambridge, MA), collagen V (ab94673; Abcam, Cambridge, MA), *α*-SMA (ab5694; Abcam, Cambridge, MA), IGF-1 (Abcam; Cambridge, MA), IGF-1R (Abcam; Cambridge, MA), and glyceraldehyde 3-phosphate dehydrogenase (GAPDH, ab9485; Abcam, Cambridge, MA). After primary incubation, the membrane was washed for 5 min (3x) in TBST before probing with secondary antibody Goat anti-Rb Alexa Fluor 568 (Life Technologies, Grand Island, NY, USA) at room temperature for 1 h with rocking. The membrane was allowed to dry before imaging using ChemiDoc-It to image. Western blots were quantified using densitometry utilizing pixels measured within each band.

### 2.5. Statistical Analysis

Statistical analyses were carried out using a 2-way ANOVA and Welch's unpaired *t*-test, calculated by GraphPad Prism 6 software. *p* < 0.05 and lower (*p* < 0.01, *p* < 0.001, etc.) were considered statistically significant. Error bars represent standard deviation. Data is representative of at least three independent experiments per donor.

## 3. Results

### 3.1. Contraction Profiles

**Figure 1:**
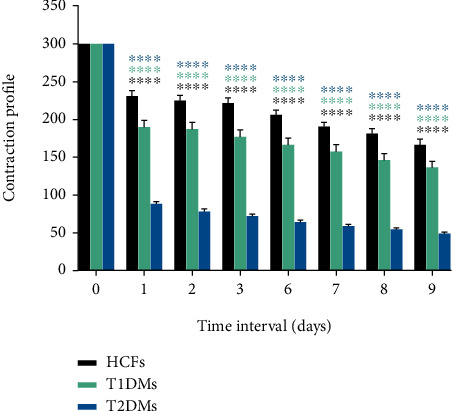
Quantification of contraction collagen matrix in HCFs, T1DMs, and T2DMs from days 0 to 9. A significant reduction is observed in the area of the collagen matrix between the three cell types correlating with increased contractility. Two-way ANOVA (*n* = 12) was used to analyze the results. Error bars represent standard deviation (∗∗∗∗ denotes *p* < 0.0001).

HCFs embedded in collagen gels maintained an average of 166.4 mm^2^ of their matrix area by day 9. On the other hand, the gel matrix area seeded with T1DMs and T2DMs only maintained an average of 136.7 mm^2^ and 48.9 mm^2^, respectively. At day 1, we identified an average of 69.3 mm^2^ reduction in gel area by HCF controls compared to a reduction of 109.9 mm^2^ in matrix area in T1DMs and 211.7 mm^2^ in T2DMs (Figure 1). By day 3, HCFs had contracted their matrix at an average rate of 26.08 mm^2^/day compared to a contraction rate of 40.97 mm^2^/day and 76.00 mm^2^/day by T1DMs and T2DMs, respectively. Our results suggest that both T1DMs and T2DMs display significantly accelerated contractility (*p* < 0.0001).

### 3.2. Corneal Fibrotic Markers

#### 3.2.1. Collagen Assembly

**Figure 2:**
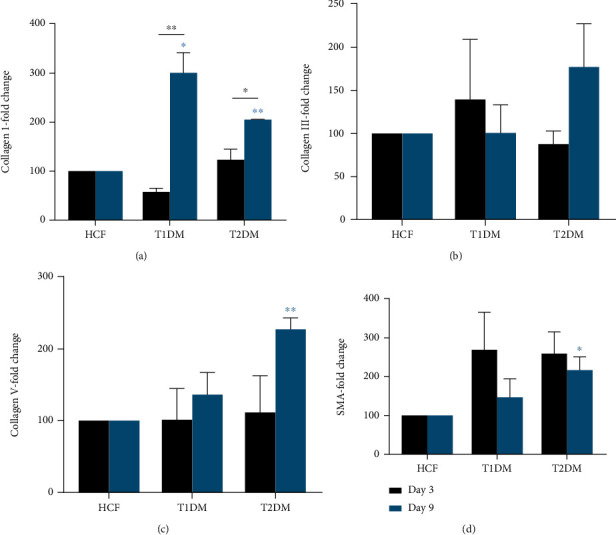
(a) Collagen I (Col I), (b) collagen III (Col III), (c) collagen V (Col V), and (d) *α*-SMA (SMA) protein levels in HCF, T1DM, and T2DM on days 3 and 9. Col I was significantly increased on day 9 in the T1DM and T2DM compared to the HCFs and between days 3 and 9. Significant increases in Col V and SMA were seen at day 9 for the T2DM when compared to HCFs at day 9. Welch's unpaired *t*-test and/or ordinary one-way ANOVA (*n* = 4) was used to analyze the results. Error bars represent standard deviation (∗∗ denotes *p* < 0.01 and ∗ denotes *p* < 0.05).

**Figure 3:**
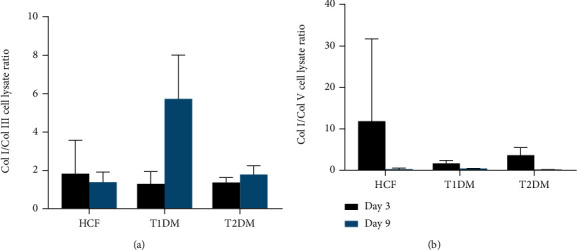
Lysate ratios of (a) Col I/Col III and (b) Col I/Col V. Welch's unpaired *t*-test and/or ordinary one-way ANOVA (*n* = 4) was used to analyze the results. Error bars represent standard deviation.

The corneal stroma is composed of various collagen types such as Col I and V, but with diseases like diabetic keratopathy, the corneal stroma composition can alter significantly. We examined protein expression in HCF, T1DM, and T2DM lysates. Initially, at day 3, there were no significant differences of Col I expression in T1DMs or T2DMs, compared to HCFs (Figure 2(a)). However, at day 9, both T1DMs and T2DMs showed significant upregulation (*p* < 0.05, *p* < 0.01), when compared to HCFs. T1DM and T2DM also showed increased expression (*p* < 0.01, *p* < 0.05) of Col I at day 9. No significant modulation of Col III expression was observed (Figure 2(b)). T2DMs showed significant upregulation (*p* < 0.01, *p* < 0.05) in both Col V and *α*-SMA, at day 9, when compared to HCFs (Figures 2(c) and 2(d)). Data analysis on both Col I/Col III and Col I/Col V ratios (Figure 3) revealed no significant changes among different cell types.

#### 3.2.2. Collagen Secretion

**Figure 4:**
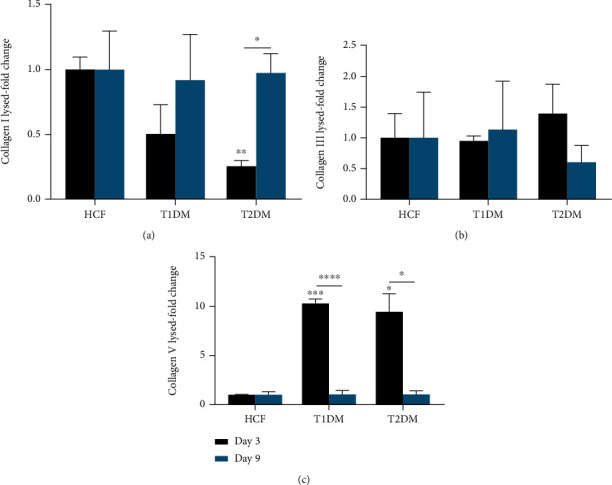
(a) Collagen I (Col I), (b) collagen III (Col III), and (c) collagen V (Col V) protein levels in conditioned media of HCF, T1DM, and T2DM on days 3 and 9. Col I expression significantly decreased on day 3 but not at day 9 compared to the HCFs. T1DM and T2DM show significant increases in Col V expression, followed by significant decreases of secretion at day 9. Welch's unpaired *t*-test and/or ordinary one-way ANOVA (*n* = 4) was used to analyze the results. Error bars represent standard deviation (∗∗∗∗ denotes *p* < 0.0001, ∗∗∗ denotes *p* < 0.001, ∗∗ denotes *p* < 0.01, and ∗ denotes *p* < 0.05).

We measured Col I, Col III, and Col V secreted into the media by HCFs, T1DMs, and T2DMs, as a function of contraction progress. Col I levels were significantly decreased (*p* < 0.05) in T2DMs at day 3, but not at day 9, when compared to HCFs (Figure 4(a)). We found a trend of upregulated Col III on T1DMs at day 9 and T2DMs at day 3; however, significance was not reached (Figure 4(b)). Col V secretion on day 3 of both T1DMs and T2DMs showed significant upregulation (*p* < 0.001, *p* < 0.05) compared to HCFs, followed by significant downregulation (*p* < 0.0001, *p* < 0.05) on day 9 (Figure 4(c)).

**Figure 5:**
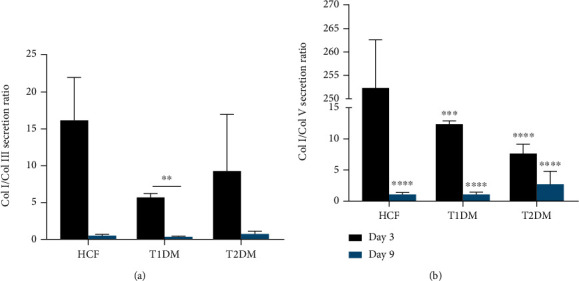
Collagen secretion ratio of (a) Col I/Col III and (b) Col I/Col V. T1DMs had a significant difference between its day 3 and 9 Col I/Col III ratio. For the Col I/Col V ratio, both days of the T1DM and T2DM showed significant decreases of secretion, as well as the HCFs at day 9. Welch's unpaired *t*-test and/or ordinary one-way ANOVA (*n* = 4) was used to analyze the results. Error bars represent standard deviation (∗∗∗∗ denotes *p* < 0.0001 and ∗∗ denotes *p* < 0.01).

Col I/Col III and Col I/Col V secretion ratios were also analyzed. There were no significant differences in the secretion ratio of Col I/Col III between the diabetic cells and healthy cells (Figure 5(a)). However, T1DMs showed a significant decrease (*p* < 0.01) of secretion between day 3 and day 9. Col I/Col V secretion was significantly downregulated (*p* < 0.001) for T1DMs and T2DMs, at both days 3 and 9, when compared to the HCFs (Figure 5(b)).

#### 3.2.3. IGF-1 and IGF-1R Protein Expression by HCFs, T1DM, and T2DM

**Figure 6:**
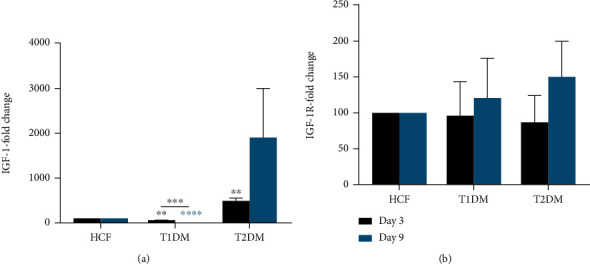
(a) Insulin-like growth factor 1 (IGF-1) and (b) insulin-like growth factor 1 receptor (IGF-1R) protein expression in HCF, T1DM, and T2DM on days 3 and 9. Welch's unpaired *t*-test (*n* = 4) was used to analyze the results. Error bars represent the standard deviation (∗∗∗∗ denotes *p* < 0.0001, ∗∗∗ denotes *p* < 0.001, and ∗∗ denotes *p* < 0.01).

We determined modulation of the key mediators in DM, insulin-like growth factor 1 (IGF-1) and its receptor (IGF-1R). Our data revealed significant upregulation (*p* < 0.01) in IGF-1 protein expression in T2DMs when compared to HCFs on day 3 (Figure 6(a)). However, IGF-1 expression was significantly downregulated at day 3 in T1DMs, including a significant decrease on day 9, indicating a possible interplay between collagen I contraction phenotype and IGF-1 DM mediator mainly in T2DM cells. IGF-1R revealed no significant changes between days 3 and 9 in HCFs, T1DMs, or T2DMs (Figure 6(b)). These results suggest that IGF-1 and IGF-1R activity might correlate with the altered contractile state of the diabetic corneal stroma.

## 4. Discussion

Diabetic keratopathy is a degenerative corneal disease observed in patients suffering from systemic DM. About 46-64% of DM patients are at risk to develop diabetic keratopathy, and with about 1 million T1DM patients in the US, diabetic keratopathy is a serious vision-threatening condition [32]. Various causes of diabetic keratopathy have been proposed, including structural abnormalities in the corneal epithelium basement membrane [33]. Studies suggested that corneal stroma, with altered/damaged basement membrane, is the reason for a delay in corneal epithelial wound healing [34, 35]. These structural changes of the basement membrane in the diabetic cornea may account for the loose attachment of corneal epithelial cells.

The corneal epithelial abnormalities in DM patients have been reported in both human and animal models. In a DM rat model of wound healing, the corneal epithelial wound closure was delayed and the phenotype of epithelium was changed [36, 37]. In addition, abnormal changes of epithelium in DM patients after cataract surgery were noted with increased average cell area and decreased hemidesmosomes. A study by Schultz et al. [13] demonstrated that corneal epithelial lesions, ranging from superficial punctate keratitis to full thickness, break in up to two-thirds of DM patients in their study [38]. The authors also reported a correlation between the severity of keratopathy and the patients' diminished peripheral sensation, suggesting that their epithelial defects and reduced corneal sensitivity are believed to be symptoms of the generalized polyneuropathy that occurs in these patients [39]. Reduced corneal sensitivity predisposes patients to corneal trauma, puts them at greater risk of developing neurotrophic corneal ulcers [40], and adversely affects corneal wound healing [41, 42].

Interestingly, high glucose was shown to independently suppress the epidermal growth factor receptor/phosphatidylinositol 3-kinase/Akt signaling pathway and altered corneal epithelial wound healing *in vitro* [43]. In addition, decreased corneal sensation and loss of specific nerve factors have been proposed as causative players in the development of diabetic keratopathy. Nakamura et al. have revealed that insulin-like growth factor 1 (IGF-1) and substance P, a neuropeptide present in sensory nerves, accelerate corneal epithelial wound healing [44]. In addition, the authors displayed that topical application of substance P and IGF-1 accelerated the corneal epithelial wound healing process in DM animals. These studies help to strengthen the potential pathogenic link between decreased corneal sensation and diabetic keratopathy. Studies by He et al. [45] revealed an entire view of the nerve architecture in human diabetic corneas. They found that decreased epithelial nerve density may result from the abnormalities of stromal nerve architecture and is affected by >5 years of T1DM. These alterations in the stromal nerves can explain the poor healing and persistent epithelial defects seen in DM patients.

The corneal stroma is also known to be affected, in diabetic keratopathy, although studies are lacking [46, 47]. The process of stromal wound healing involves wound contracture, bringing the margins of open wounds together [48]. Mechanical forces generated by fibroblast cells lead to wound contraction and potentially tissue scarring. The response of corneal keratocytes to growth factors secreted postinjury can also be modulated by changes in ECM stiffness. Several studies [49] have demonstrated that fibroblast growth factor-2 (FGF2) induces fibroblast transformation of keratocytes on rigid 2D substrates, as shown by changes in cell morphology and development of stress fibers and focal adhesions [50]. However, within hydrated 3D collagen matrices, FGF2 stimulates ruffling of keratocyte processes without inducing major changes in cell morphology, formation of stress fibers, or collagen matrix organization [51]. When corneal keratocytes are seeded within compressed 3D collagen matrices, fibroblast transformation is again observed [52]. Various studies are aimed at understanding the mechanisms involved in tissue contraction after corneal stromal wounding; Hibino et al. showed rabbit keratocytes cultured in a collagen gel, which contracted in the presence of Fetal Calf Serum (FCS) [53]. These findings highlight the fact that in corneal wound healing, the epithelial cells and keratocytes modulate cellular activities facilitating epithelial migration. In addition, Andresen et al. also utilized collagen gels to demonstrate that the composition of the ECM influences the motility of the human corneal fibroblasts, either through cell-matrix or matrix-matrix interactions, thus facilitating entry of migrating keratocytes into the wound [54].

In this study, we assessed the diabetic corneal fibroblast behavior utilizing 3D collagen matrices. DM corneal cells demonstrated structural interactions with collagen fibrils, resulting in contraction of the gel matrices. By utilizing preassembled 3D collagen gels seeded with HCFs, T1DM, and T2DM cells, changes in contraction rate were measured over a period of 9 days and compared to healthy controls. DM cells showed increased contractility correlated with specific corneal fibrotic markers.

Assessment of overall ECM contraction is a valuable assay for assessing DM changes in cell contractility compared to HCFs. Our data revealed that mainly T2DM and to a lesser extent T1DM display a significantly accelerated contractility of the collagen gel matrix and altered collagen I and V expression compared to HCFs. Contraction of the ECM is important in normal wound healing processes within the cornea. The accelerated contraction profile exhibited by DM cells suggests that stromal fibroblasts in DM corneas are responding to external stimuli, perhaps overreacting leading to more scarring/fibrosis. Depending on the balance of DM mediators, the extent of the wound, and the duration of a hyperglycemic state, the outcome of stromal healing can be regeneration of normal stromal structure or an opaque scar. Our collagen gel model mirrors an *in vivo* DM corneal stroma and allows the dissection of the stromal environment and its resident cells during ECM remodelling and wound repair. Thus, our 3D collagen gel model allowed us to simulate a stromal *in vivo* environment and study the stromal wound healing process.

## 5. Conclusions

Our data supports DM keratopathy as a stressor of ECM remodelling generating large contractile forces, both of which can alter corneal clarity and result in corneal scarring. In human DM cells, our study revealed abnormal overexpression of insulin-like growth factor (IGF) 1 and suggests that diabetic keratopathy is a result of decreased migratory growth factor levels that could lead to BM degradation and clinically observed delayed wound healing compared with normal cells. The finding confirms that the mechanical properties of collagen matrices should be considered in the cellular contraction events induced by DM keratopathy. Overall, the study has shown the importance of matrix properties in the design of collagen-based biomaterial for clinical applications.

## Data Availability

Data will be available upon request from the corresponding author. Please contact Dr. D. Karamichos (dimitrios.karamichos@unthsc.edu).
